# Impacts of gravels on the moisture evaporation and desiccation cracks in soil

**DOI:** 10.3389/fchem.2026.1964498

**Published:** 2026-09-28

**Authors:** Zhenghui Li, Juntao Bai, Xinwen Zhao, Ao Zhang, Jun He, Chen Chen

**Affiliations:** 1 College of Civil Engineering and Architecture, China Three Gorges University, Yichang, China; 2 Wuhan Center, China Geological Survey, Ministry of Natural Resources (Geosciences Innovation Center of Central South China), Wuhan, China

**Keywords:** clay, desiccation crack, geometric morphology, gravels, moisture evaporation

## Abstract

Under extreme arid conditions, soil loses moisture and forms cracks, which significantly affect their mechanical and hydraulic properties (e.g., shear strength, soil structure, and hydraulic behavior). Natural soils commonly contain gravels. However, research on the effects of gravels on moisture evaporation and desiccation cracking in soil is limited. To investigate the influence of gravel content on desiccation cracking behavior, soil samples were prepared by mixing clayey soil with quartz sand (particle size predominantly between 2 mm and 5 mm). Desiccation cracking experiments were performed in a custom-built chamber with controlled temperature and humidity, equipped with an automated real-time imaging system. Computer-based image processing techniques were employed to quantitatively describe the cracking characteristics. It was found that under evaporation conditions, gravels in soil hinder the upward migration of internal moisture to the surface. This obstruction leads to the formation of menisci within surface soil pores, generating tensile stress between soil particles due to surface tension. Additionally, the presence of gravels causes uneven settlement in the soil during evaporation, resulting in additional tensile stress between particles. As a result, compared to pure clayey soil, gravels alter the tensile stress field within the soil. This alteration not only accelerates crack initiation but also shifts the initial crack location and modifies the final geometric characteristics of the cracks. Conversely, gravels inhibit the vertical growth of cracks and the lateral shrinkage of soil blocks, thereby reducing overall crack density on the soil surface. This study offers a new perspective for interpreting the mechanisms of desiccation cracking in gravel-containing clay under natural conditions.

## 1 Introduction

In recent years, extreme drought events have become more frequent, intense, prolonged, and widespread globally. Drought significantly alters the spatiotemporal distribution of moisture in soil, which in turn changes the soil’s engineering properties. In arid conditions, moisture evaporation causes clay to shrink and develop cracks, which significantly affect mechanical and hydraulic properties of soil (Morris et al., 1992; Tang et al., 2010; Chaduvula et al., 2017). Both experimental findings (Rayhani et al., 2007; Cheng et al., 2021) and field monitoring (Xu et al., 2024a; Xu et al., 2024b; Zhang et al., 2024) demonstrate that desiccation cracks in soil can significantly enhance soil permeability by their dual porosity behaviour. Studies conducted by Kumar et al. (2024) and Luo et al. (2025) have elucidated that the strength degradation induced by desiccation cracking—manifested as irreversible macroporosity and skeletal damage—fundamentally overrides the hardening effect of matric suction, thereby driving a cumulative and critical attenuation in bulk shear strength. Additionally, the presence of desiccation cracks in soil directly or indirectly affects the geological structure (Tang et al., 2012; Jones et al., 2014; Zhang et al., 2016; Cheng et al., 2020). For instance, vertical cracks directly compromise the integrity of the soil mass by creating dynamic discontinuities. Indirectly, these fissures serve as preferential flow channels that bypass the surface matrix, rapidly transmitting hydrostatic pressure to deeper strata. This process shifts the potential failure surface from a shallow wetting front to a deep-seated composite sliding zone, fundamentally altering the slope’s structural stability mechanism (Luo et al., 2025). Studies have shown that cracks can lead to landslides, instability in foundations, embankments, earth dams, and other structures related to cohesive soil (Lozada et al., 2015; Jamalinia et al., 2020; Stirling et al., 2021; Chen et al., 2025). The escalating frequency of severe droughts has led to a growing focus on the issue of desiccation cracking in cohesive soil within the academic community.

Investigating the desiccation cracks process is a crucial part of understanding the mechanism of crack initiation and development under drought condition. In terms of monitoring and detecting techniques of the soil desiccation cracking, surface photography (Cai et al., 2023), laser microscopy (Uday and Singh, 2013a), 3D scanner system (Zhuo et al., 2024), dual-probe heat-pulse technique (Palaparthy et al., 2018), infrared thermal imaging technology (Lin et al., 2021), X-ray computed tomography (Zeng et al., 2024), optical fiber sensing (Liu et al., 2024) or electrical resistivity method (Tang et al., 2018) have provided a lot of details for drying-induced clayey soil desiccation cracking process in laboratory or field. With these experimental setups and techniques, Various factors influencing the soil moisture evaporation and the response of soil deformation and cracking were explored, which include temperature and humidity (Tang et al., 2010; Uday and Singh, 2013b; Uday et al., 2015), drying-wetting cycles (Zhuo et al., 2024; Wanare et al., 2021), solvent (Khatun et al., 2015; Zhang et al., 2019), specimen geometry (Lakshmikantha et al., 2012; Lakshmikantha et al., 2018), soil layer thickness (Tang et al., 2007), roughness of substrate (DeCarlo and Shokri, 2014; Zeng et al., 2019).

In its natural state, soil is a heterogenous material, consisting not only of particles smaller than 2 mm in diameter, but also of coarser fractions such as gravel, which is defined by a particle diameter exceeding 2 mm according to Chinese standard for engineering classification of soil (GB/T 50145-2007). However, previous studies predominantly used soils passed through a 2 mm sieve as test materials in soil desiccation cracking experiments (Tang et al., 2010; Tang et al., 2011; Cai et al., 2023). Investigating the effect of gravel on the desiccation cracking of soil is therefore critical for accurately representing field conditions. Previous studies have established that sand particles (diameter <2 mm) influence the shrinkage behavior of cohesive soils. For example, Cui et al. (2012) observed that the shrinkage potential of Gaomiaozi expansive soil–sand mixtures decreased with increasing sand content. In contrast, the impact of gravel on moisture evaporation and desiccation crack evolution in soil remain poorly understood. The objective of this research is to investigate the effects of gravels on desiccation cracking in soil. Quartz sand with a dominant particle diameter exceeding 2 mm was employed to simulate gravel in the drying experiments. The impact of gravel on evaporation-induced desiccation cracking was quantitatively investigated using image processing techniques, with a focus on the geometric morphology of the surface crack networks. This study aims to enhance the mechanistic understanding of how the presence of gravel influences soil desiccation cracking.

## 2 Materials and methods

The clayey soil utilized in this study was from Badong area in Hubei Province, China and the silica sand was used to simulate the gravels with the size ranged from 4–6 meshes. Their properties are listed in Table 1. The soil samples were prepared in three procedures according to previous studies (Tang et al., 2010; Tang et al., 2012). First, the clayey soil was air dried, crushed and sieved to 2 mm. Two buckets were prepared for the pure clayey soil and clayey soil-gravel mixture in which the volume of silica sand accounted for 40% of the mixture’s grains. Secondly, the distilled water was added into the two buckets and mixed thoroughly at the water content of about 150%. Then they were poured into two 20 cm-diameter and 2 cm-hight glass plates, respectively and were placed on a vibration device for 5 min to remove the air bubbles (Tang et al., 2021). Finally, the two glass plates were covered with a plastic membrane for 3 days in order to make the moisture as uniform as possible and allow sedimentation of slurry. After the 3-day sedimentation period, the final specimen thickness was approximately 1.5 cm for both samples, corresponding to a total specimen volume of about 471 cm^3^. For the pure clay specimen, the dry clay mass was 350 g, yielding a dry density of the clay fraction of 0.74 g/cm^3^. For the clayey soil–gravel mixture, the dry clay mass was 315.75 g and the quartz sand mass was 122.20 g. Given a specific gravity of 2.65, the volume occupied by the quartz sand was 46.1 cm^3^; the remaining volume of 424.9 cm^3^ was occupied by the clay fraction, which also had a dry density of 0.74 g/cm^3^. Thus, the clay matrix in both specimens had essentially the same initial dry density and physical state.

**TABLE 1:** Properties of clayey soil and silica sand.

Material	Properties		Value
Clayey soil	Specific gravity		2.74
	Liquid limit		43.86%
	Plastic limit		25.98%
	Plasticity index		17.9
	Soil classification (GB/T 50145-2007)		CL
	Particle size	0.075 mm–2 mm	13.86%
		0.005 mm–0.075 mm	56.55%
		<0.005 mm	29.59%
Silica sand	Specific gravity		2.65
	Particle size	5 mm–7 mm	1.34%
		2 mm–5 mm	97.94%
		1 mm–2 mm	0.09%
		<1 mm	0.62%

Prior to the test, the supernatant water above the settled slurry surface was suctioned out as much as possible by using a syringe for reducing the experimental duration, because its evaporation has a negligible effect on soil fabric shrinkage. Due to its significantly greater density compared to clayey soil, the quartz sand is considered to have negligible water storage capacity. Consequently, the analysis in this study focuses exclusively on the water content of the clayey soil fraction in the pure clayey soil and the clayey soil-gravel mixture specimens. At the beginning of the experiment, the water content of clayey soil fraction in these two specimens were 77.19% (pure clayey soil) and 91.03% (clayey soil-gravel mixture), respectively. It should be noted that the higher calculated water content in the clayey soil–gravel mixture specimen was an artifact of the preparation procedure. During removal of the supernatant water with a syringe, a slightly thicker layer was deliberately left above the settled mixture surface to avoid any disturbance from the needle tip. In the pure clay specimen, the supernatant was removed more completely. This difference does not affect the comparisons in this study, because all water contents are expressed relative to the clay fraction only, and the clay matrix in both specimens had essentially the same initial density and physical state.

It is worth noting that the higher calculated water content in the clayey soil-gravel mixture specimen stems primarily from a greater retention of supernatant water after the initial preparation step. Following the procedures described above, however, the actual state (refers to the initial fabric, density, void ratio, and degree of saturation of the clay matrix) of the clayey soil fraction were essentially the same in both the pure clayey soil specimen and clayey soil-gravel mixture specimen.

Since the primary aim of this study was to investigate the effect of gravel on moisture evaporation and desiccation cracking, a representative and more commonly encountered temperature was selected as a controlled environmental parameter, rather than testing a series of temperatures. During desiccation, these two specimens were placed in a constant temperature and humidity chamber at temperature 35 °C ± 1 °C and at relative humidity (RH) 55 ±2 %, as shown in Figure 1. Meantime, the specimens were weighed to an accuracy of 0.01 g every 3 min in order to record the water loss at varying intervals and the corresponding water content can be determined. The constant temperature and humidity chamber was equipped with white LED lights mounted above and to the sides of the specimens to provide uniform illumination, avoiding shadows and reflections. The digital cameras were fixed above the specimens to monitor cracks development by acquiring images, while the lighting conditions were kept constant throughout image acquisition. The images were used to determining the geometric parameters of cracks, such as crack area, width, length etc. Since the crack images captured by the digital camera are in RGB color, they cannot be directly used for quantitative analysis for crack geometric morphology. It is necessary to binarize these color images to obtain binary crack images (Figure 2). These binary images are then subjected to quantitative analysis. The detailed procedure of digital image processing can be found from Tang et al. (2008).

**FIGURE 1:**
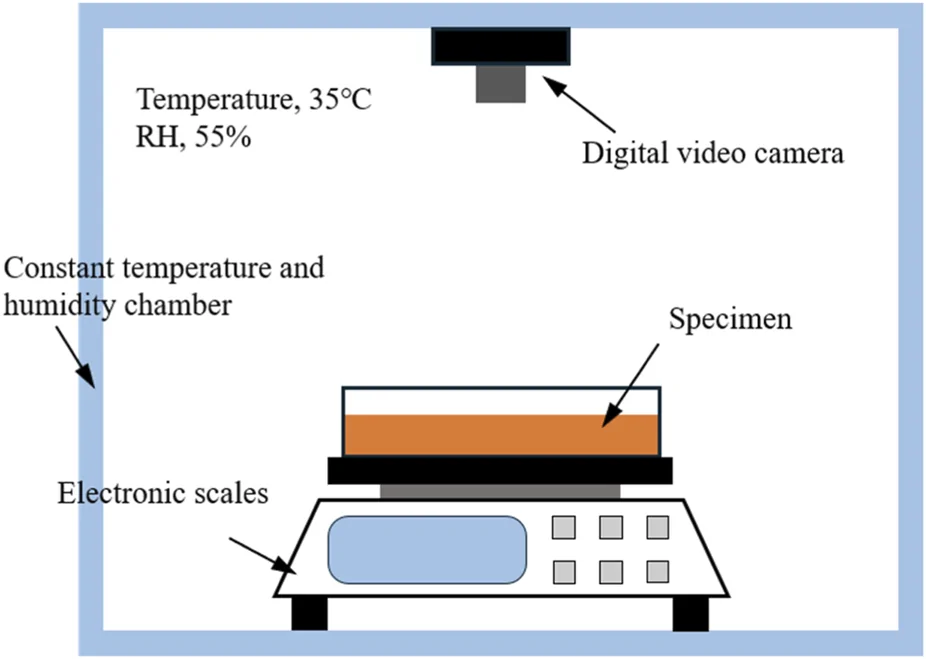
Schematic of experimental set-up used for moisture evaporation and desiccation crack observation.

**FIGURE 2:**
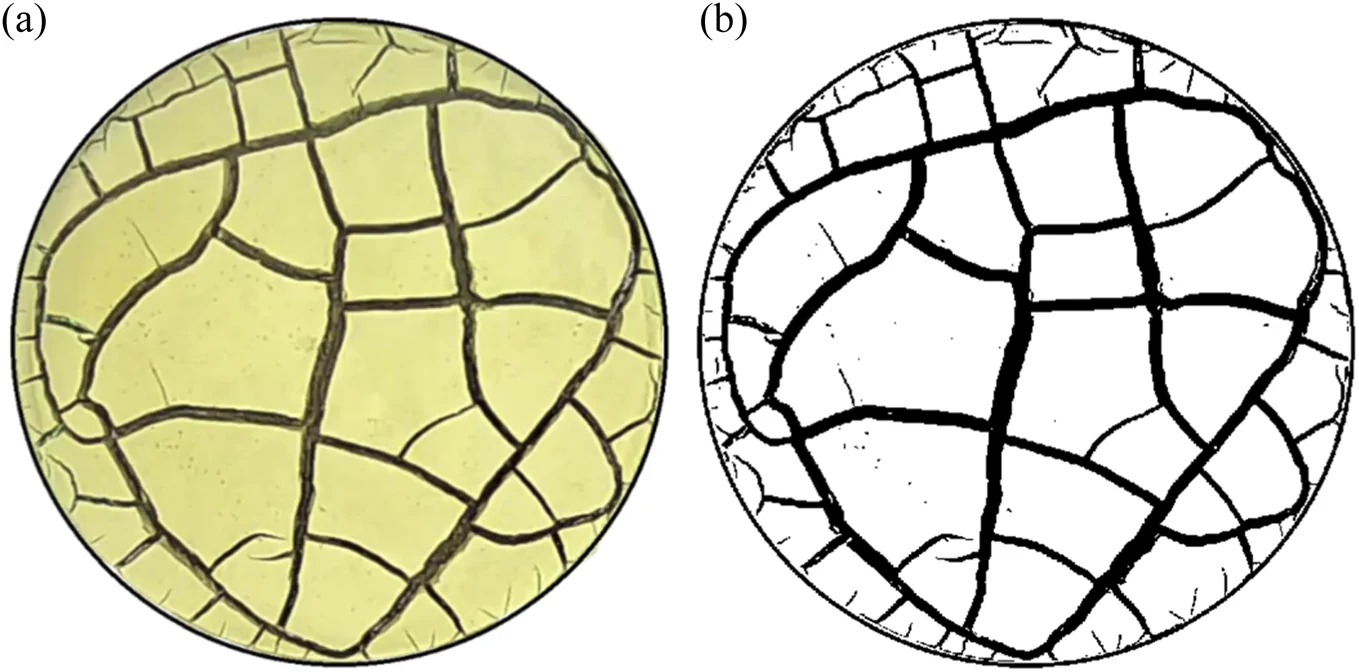
Digital image processing: **(a)** RGB image, **(b)** Binary image.

## 3 Results

### 3.1 Evaporation process

Figure 3 presents the evolution of both the water content ( w ) of the clayey soil fraction and the evaporation rate ( $R_{e}$ , defined as the mass of water loss per minute) with elapsed drying time for the two specimens. During drying, the evaporation rate for both specimens transitioned from a constant phase to a falling phase, ultimately declining to zero. During the initial stage, the water content of the clayey soil fraction in both specimens decreased linearly with time. In this constant-rate stage, the evaporation rates remained approximately constant at 0.107 g/min for the pure clayey soil and 0.101 g/min for the clayey soil-gravel mixture. The near-identical evaporation rates in this stage are attributed to both specimens being initially saturated, which maintains a relatively constant vapor pressure at the soil-pore water interface. Once the water content decreased to that corresponding to the air entry value ( $w_{AE}$ ) where the specimen starts to desaturate, both the water content and the evaporation rate declined steadily until reaching zero at the residual stage. Note that in this study, the air entry value is defined as the water content at which the evaporation rate begins to decrease from the constant-rate stage, indicating that air starts to enter the soil pores and the specimen transitions from a saturated to an unsaturated state. It was determined directly from the measured evaporation curves.

**FIGURE 3:**
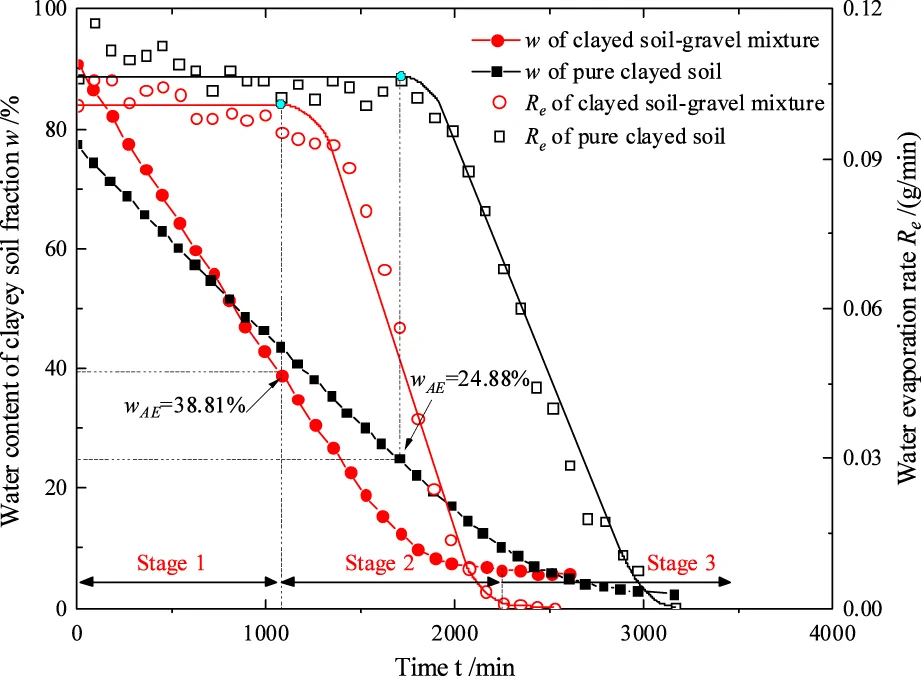
Evolution of water content and evaporation rate over time.

### 3.2 Desiccation cracks initiation and propagation

The evolution of crack patterns of pure clayey soil and clayey soil-gravel mixture, as water content decreases, are presented in Figures 4, 5, respectively. Cracks in pure clayey soil initiated at the specimen’s edge when the water content w was 47.81%, and they propagated towards the opposite edge as water continued to evaporate (Figures 4A_1_–B_1_). New cracks tended to form as straight lines (“—”), starting at the specimen’s center and terminating when they joined existing cracks or reached the edge (Figures 4C_1_–F_1_). When the water content w dropped below 28.54%, no new cracks formed, instead, existing cracks widened. (Figures 4F_1_–H_1_).

**FIGURE 4:**
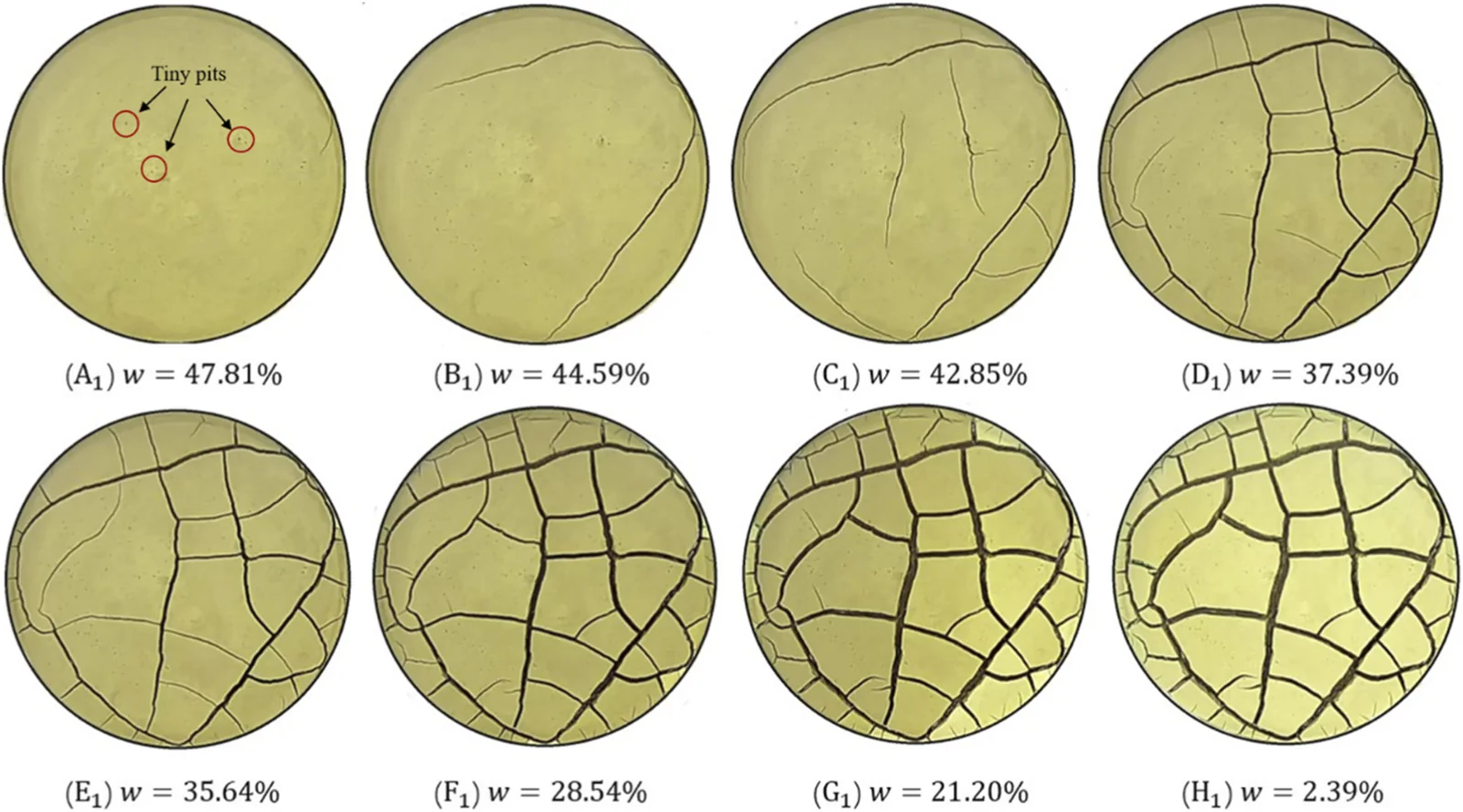
Evolution of surface crack patterns in the pure clayey soil specimen versus decreasing water content: **(A1)** w=47.81%; **(B1)** w=44.59%; **(C1) **w=42.85%; **(D1)** w=37.39%; **(E1)** w=35.64%; **(F1)** w=28.54%; **(G1)** w=21.20%; **(H1)** w=2.39%.

**FIGURE 5:**
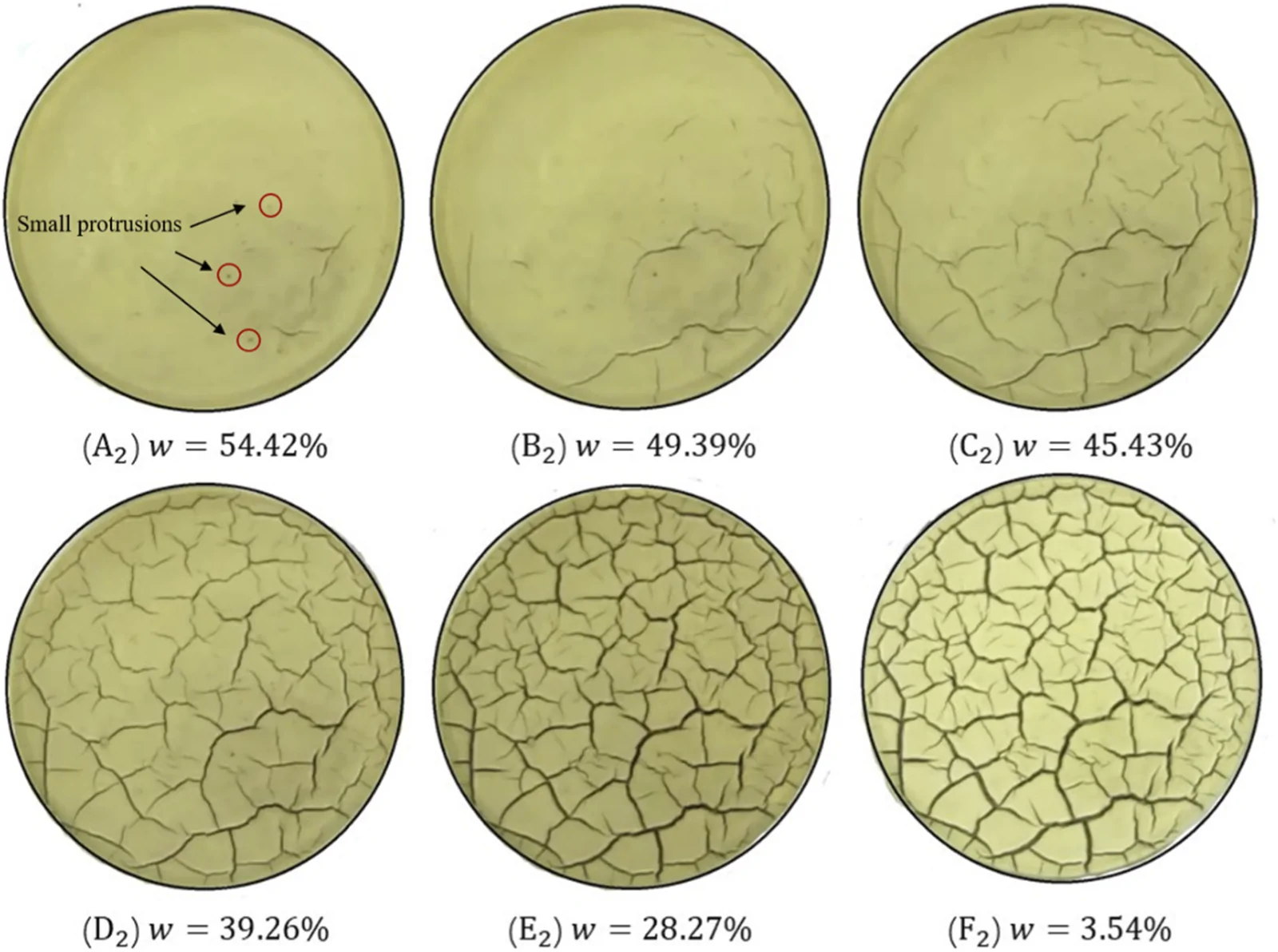
Evolution of surface crack patterns in the clayey soil-gravel mixture specimen versus decreasing water content: **(A2)** w=54.42%; **(B2)** w=49.39%; **(C2)** w=45.43%; **(D2)** w=39.26%; **(E2)** w=28.27%; **(F2)** w=3.54%.

However, observations of the crack pattern evolution in the clayey soil-gravel mixture reveal differences in both the initiation position and the forms of the cracks compared to pure clayey soil. Cracks in the mixture first appeared at the center and the edge of the specimen when the water content w was 54.42% (Figure 5A_2_). These cracks tended to form “T” or “Y” shapes and terminated upon joining other cracks or reaching the edge (Figures 5B_2_–E_2_). Similar to pure clayey soil, when the water content w decreased to 28.27%, this stage mainly involved the widening of existing cracks until desiccation ceased (Figures 5E_2_–F_2_). Compared to the pure clayey soil’s crack patterns at the end of drying, the mixture exhibited a higher number of cracks, narrower crack widths (see also Figure 6), and more uniform pattern density in fracture geometry.

**FIGURE 6:**
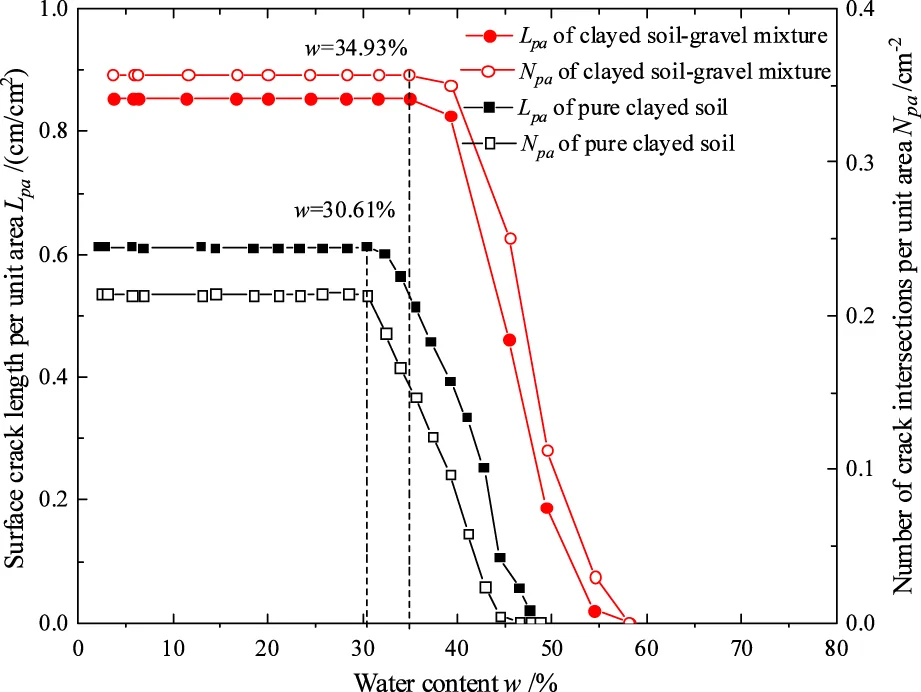
Surface crack length per unit area *L*_pa_ and number of surface crack segment per unit area *N*_pa_ of the pure clayey soil and clayey soil-gravel mixture with decreasing water content.

### 3.3 Crack geometric morphology

Figure 7 illustrates how the surface crack ratio $R_{C}$ , defined as the ratio of crack surface area to total specimen surface area, changes with water content w during drying. It is observed that as the water content decreases to the cracking water content $w_{C}$ , which is 48.53% and 58.16% for pure clayey soil and clayey soil-gravel mixture, respectively, the crack ratio $R_{C}$ of these two specimens increases rapidly. However, as the water content w dropped below the air entry value $w_{AE}$ , the increase rate of $R_{C}$ significantly decreased, especially for pure clayey soil. Eventually, $R_{C}$ reaches zero when the water content decreases to the shrinkage limit $w_{SL}$ . The surface cracks of specimens can be categorized into two types: those appearing before reaching the $w_{AE}$ and those appearing after reaching the $w_{AE}$ . Figure 7 shows that the area of surface cracks appearing before reaching the $w_{AE}$ accounts for 83.72% in pure clayey soil and 50.43% in clayey soil-gravel mixture. The cracking water content $w_{C}$ of clayey soil-gravel mixture (58.16%) is higher than that of pure clayey soil (48.53%). Additionally, the increase rate of $R_{C}$ in pure clayey soil was faster than that in clayey soil-gravel mixture. In contrast, at the end of evaporation, the $R_{C}$ of pure clayey soil (20.21%) is 1.23 times greater than that of clayey soil-gravel mixture (16.42%).

**FIGURE 7:**
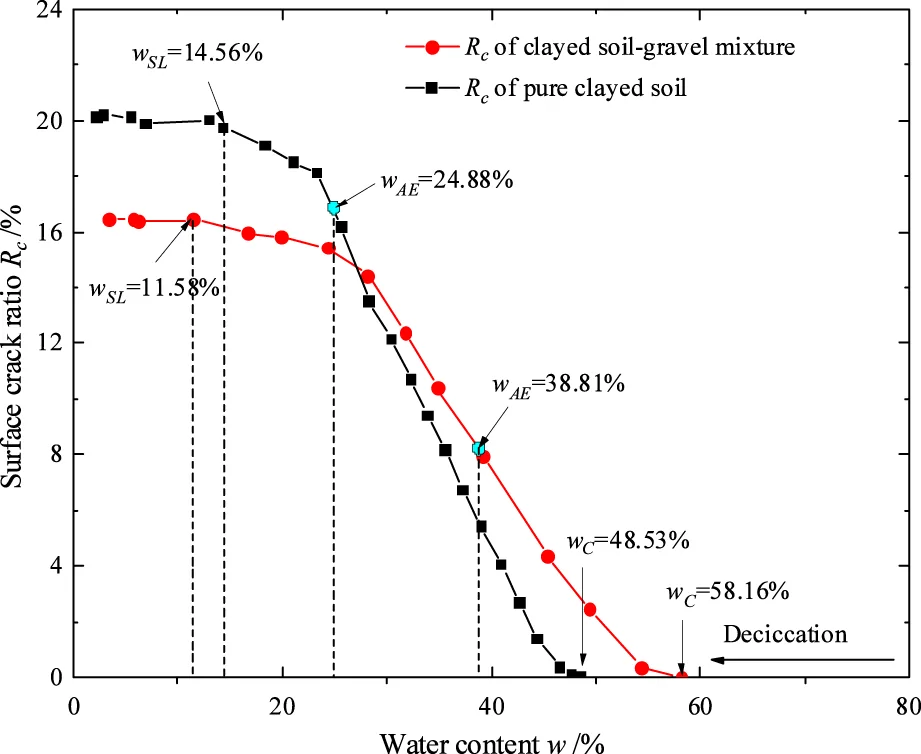
Surface crack ratio $R_{C}$ of the pure clayey soil and clayey soil-gravel mixture with decreasing water content.

The evolution of the average surface crack width ( $W_{a}$ ) with water content during drying is presented in Figure 8 for both the pure clayey soil and the clayey soil-gravel mixture. The average surface crack width $W_{a}$ is defined as the ratio of the total surface area of the cracks to their cumulative length. When the water content was at the range from 40% to 60%, the average surface crack width did not vary monotonically with the decrease of water content. The average surface crack width increased quick with water content reducing after the water content is less than 40%. Then the average surface crack width maintained a stable value after the water content was closed to the shrinkage limit. The subsequent value of the pure clayey soil’s $W_{a}$ (3.28 mm) was about 2.3 times larger than that of the clayey soil-gravel mixture (1.92 mm).

**FIGURE 8:**
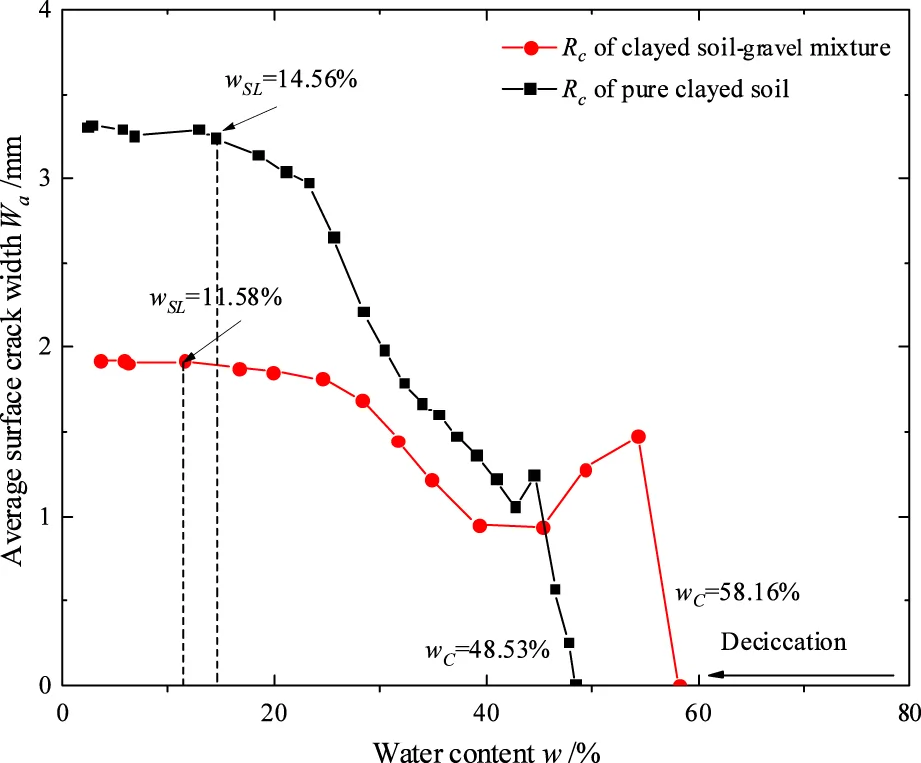
Average surface crack width $W_{a}$ of the pure clayey soil and clayey soil-gravel mixture with decreasing water content.

Figure 6 depicts $L_{pa}$ , the surface crack length per unit area, and $N_{pa}$ , the number of surface crack segments per unit area, as they vary with water content during drying. To some extent, $L_{pa}$ and $N_{pa}$ can reflect the degree of cracking of the soil specimen surface (Tang et al., 2011). There was no new intersection points initiated nor an increase in crack length when the water content of specimens reached 30.16% (pure clayey soil) and 34.93% (clayey soil-gravel mixture), respectively. Ultimately, the $L_{pa}$ of clayey soil-gravel mixture (0.85 cm/cm^2^) was approximately 1.39 times that of the pure clayey soil (0.61 cm/cm^2^). The $N_{pa}$ of the clayey soil-gravel mixture (0.36/cm^2^) was 1.71 times higher than that of pure clayey soil (0.21/cm^2^).

## 4 Discussion

### 4.1 Influence on moisture evaporation

Hillel (1982) stated that evaporation from soils requires the vapor pressure at hydro-pneumatic (viz. water-air) interfaces to exceed that of the ambient air. The rate and duration of water evaporation depend on factors including temperature, relative humidity, wind velocity, solar radiation, soil suction, salt concentration, and soil pore size. Generally, the evaporation process can be classified into three stages based on the rate of evaporation:

Stage 1: Under constant drying conditions, water evaporation initiates from the supernatant of an initially fully saturated slurry. During this phase, evaporation minimally affects the shrinkage of the soil fabric (refer to Figure 3). Neglecting the saline ions from the soil in the supernatant water, the vapor pressure above the supernatant water closely approximates the saturated vapor pressure $v_{v0}$ under drying conditions. Once the water-air interface reaches the surface layer, the specimen begins normal shrinkage but remains saturated for a period (Stirk, 1954; Tang et al., 2011). This is because the specimen’s volume decrease equaled the volume of water loss during this phase. Consequently, during this stage, the evaporation rate maintains a relatively constant value due to consistent vapor pressure at hydro-pneumatic interfaces.
Stage.2: When the water content decreased to the air-entry value $w_{AE}$ during further drying, the specimen’s volume decrease became less than the volume of water loss. This marks the onset of residual or zero shrinkage. As a result, the saturated slurry becomes unsaturated as air enters the soil pore. During this phase, a water-air meniscus is formed between soil particles. The curvature of the capillary meniscus increased as water content decreased, leading to a reduction in vapor pressure at the meniscus. Due to the gradual reduction in the vapor pressure gradient across the soil-air interface, the evaporation rate decreased progressively during this stage. Because the specimen remains essentially saturated during the constant-rate stage and volume decrease is approximately equal to water loss, $w_{AE}$ also marks the transition from the saturated shrinkage stage to the unsaturated stage in which tensile stresses and desiccation cracks develop.
Stage 3: When the curvature of the capillary meniscus reaches a constant value under constant drying conditions, the vapor pressure at the water-air meniscus becomes equal to the atmospheric vapor pressure. This equilibrium does not imply that no moisture escapes from the soil to the atmosphere. During this period, the amount of moisture escaping from the soil to the atmosphere equals the amount entering the soil from the atmosphere. As a result, the evaporation rate becomes zero.

Both the pure clayey soil and the clayey soil-gravel mixture underwent the three stages described above. However, a distinct difference was observed: the water content at the air-entry value ( $W_{AE}$ ) differed significantly between the two specimens. This difference can be explained with reference to Figure 9, a schematic illustrating moisture transport within the soils (arrows indicate the direction of moisture movement). Compared to the pure clayey soil, the presence of gravel—a relatively impermeable material—fundamentally alters moisture transport paths in the mixture. This alteration increases the travel distance for moisture and reduces the effective cross-sectional area available for flow. According to Tang et al. (2011), the decrease in evaporation rate can be attributed to several factors: decreasing water availability in the soil specimens over time; increasing suction that binds water molecules and prevents their escape to the atmosphere; and decreasing vapor pressure gradient across the soil-air interface during drying. The presence of gravel reduces the availability of water at the soil surface, leading to a lower value of vapor pressure $v_{vm}$ compared to that of the pure clay $v_{vc}$ . By hindering moisture transport, the gravels impede upward water movement. This may result in a localized reduction in moisture supply to the soil surface above or around gravel inclusions, leading to a non-uniform moisture distribution in the soil profile. This interpretation is consistent with the reduced evaporation rate and higher air-entry water content observed in the mixture, although direct evidence of the local moisture distribution was not obtained in this study. The transition from saturated to unsaturated conditions at the soil surface occurs when the evaporation rate exceeds the rate of moisture supply from the underlying soil. This mechanism explains why the surface of the saturated clayey soil-gravel mixture becomes unsaturated at a higher overall water content than that of the pure clayey soil.

**FIGURE 9:**
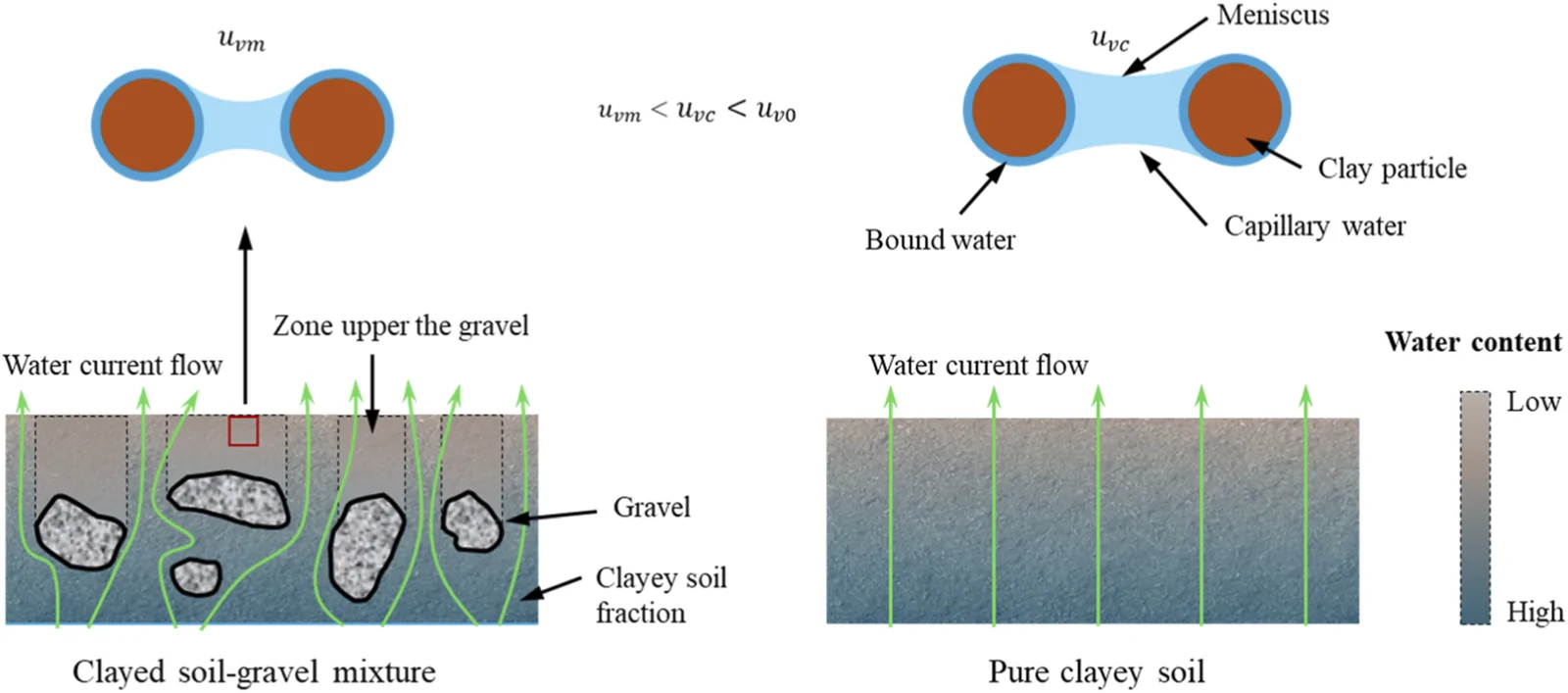
Effects of gravels on the moisture evaporation of soil.

### 4.2 Influence on desiccation crack development

Generally, cracks initiate and develop primarily due to tensile stress caused by soil dehydration and shrinkage (Uday et al., 2014; Shinde et al., 2012). A layer of bound water film surrounds the clay mineral surface under the hydration effect of cations adsorbed on the surface of clay mineral crystal layers. Under fully saturated, low-salinity conditions and at relatively low effective stress, clay particles in a dispersed state are separated by surface forces governed by the electrical double layer (EDL). The negatively charged surfaces of clay minerals attract exchangeable cations and polar water molecules, forming a diffuse ionic layer. When adjacent particles approach, repulsive forces associated with overlapping EDLs and, at very small separations, hydration forces can balance van der Waals attraction, thereby maintaining a finite separation distance (Mitchell and Soga, 2005). This equilibrium spacing, which depends on the EDL thickness and the balance of interparticle forces, contributes to the initial porosity and provides the potential for subsequent shrinkage as water is removed or effective stress increases. This spacing provides substantial capacity for soil shrinkage upon water loss. During the drying process, free water in the pores of the surface begins to evaporate initially. Continued evaporation generates capillary action in the surface soil, continuously transferring water from lower layers to the upper water-air interface to sustain evaporation. Capillary action generation is linked to surface tension of pore water, causing the liquid surface at the water-air interface to bend. The menisci’s surface tension applies shrinkage stress to the soil particles in both lateral and vertical directions. Consequently, soil particles move closer horizontally and consolidate vertically, reducing soil volume and pore spaces. Each particle experiences shrinkage stress from neighboring particles in various directions, forming a tensile stress field within the soil.

During the drying process, as tensile stress between soil particles gradually increases, cracks form when this stress exceeds the soil particles’ tensile strength (Jayanthi et al., 2017). At this point, the accumulated strain energy in the soil is released, causing the stress field to readjust. The bonding strength between soil particles exhibits inherent variability due to the heterogeneous nature of soil, which encompasses compositional complexity, structural anisotropy, and environmental sensitivity. Consequently, the distribution of the tensile stress field in the soil is uneven, leading to stress concentration and crack formation in areas where the bonding strength is weak and deformation is restricted. For instance, extensive investigations (Baer et al., 2009; Tang et al., 2021) indicate that cracks typically initiate at small surface pits characterized by stress concentration and shrinkage distortion. Cracks also tends to initiate at the specimen’s edges possibly due to defects or boundary effects, with additional heat transfer occurring at the specimen-container interface. After the soil is divided into blocks by cracks, the cracks gradually widen and deepen as the adjacent soil blocks shrink. As drying progresses, this process continues, leading to further shrinkage and densification of the soil blocks until the soil particles come into contact and the shrinkage ceases.

The experimental results indicate that gravels increase the water content at the onset of soil cracking and significantly alter crack geometry, resulting in more, longer, and narrower cracks. Figure 10 illustrates the effects of gravels on the initiation and development of desiccation cracks. As water evaporates, the surface moves vertically, accompanied by differential settlement. Because gravels do not shrink like clayey soil, this causes more small protrusions on the specimen surface, inducing additional tensile stress. The tensile stress developing during drying originates from two primary sources: capillary suction due to meniscus formation and an additional component induced by differential settlement (Figure 10b). Greater differential settlement occurs in the clayey soil-gravel mixture, thereby generating higher additional tensile stress compared to the pure clayey soil. Consequently, at an identical water content of the clayey soil fraction, the total tensile stress is higher in the clayey soil-gravel mixture than in the pure clayey soil. This higher total tensile stress leads to earlier cracking, explaining why the critical water content for crack initiation is higher in the clayey soil-gravel mixture. When the tensile stress exceeds the tensile strength between soil particles, cracks appear on the soil surface. According to fracture mechanics, stress concentration at the crack tips leads to downward propagation until the cracks are blocked, while the specimen also shrinks laterally. However, gravels can impede lateral widening and downward propagation of cracks, as shown in Figure 10c. When crack tips encounter gravels, downward propagation stops. As a result, the crack depth decreases, as does the width, because crack width is closely related to crack depth. After the initial cracks divide the clayey soil-gravel mixture into blocks, the shrinkage of these blocks is limited by the approach and connection of gravels. Consequently, the crack width in the clayey soil-gravel mixture is significantly narrower than in pure clayey soil.

**FIGURE 10:**
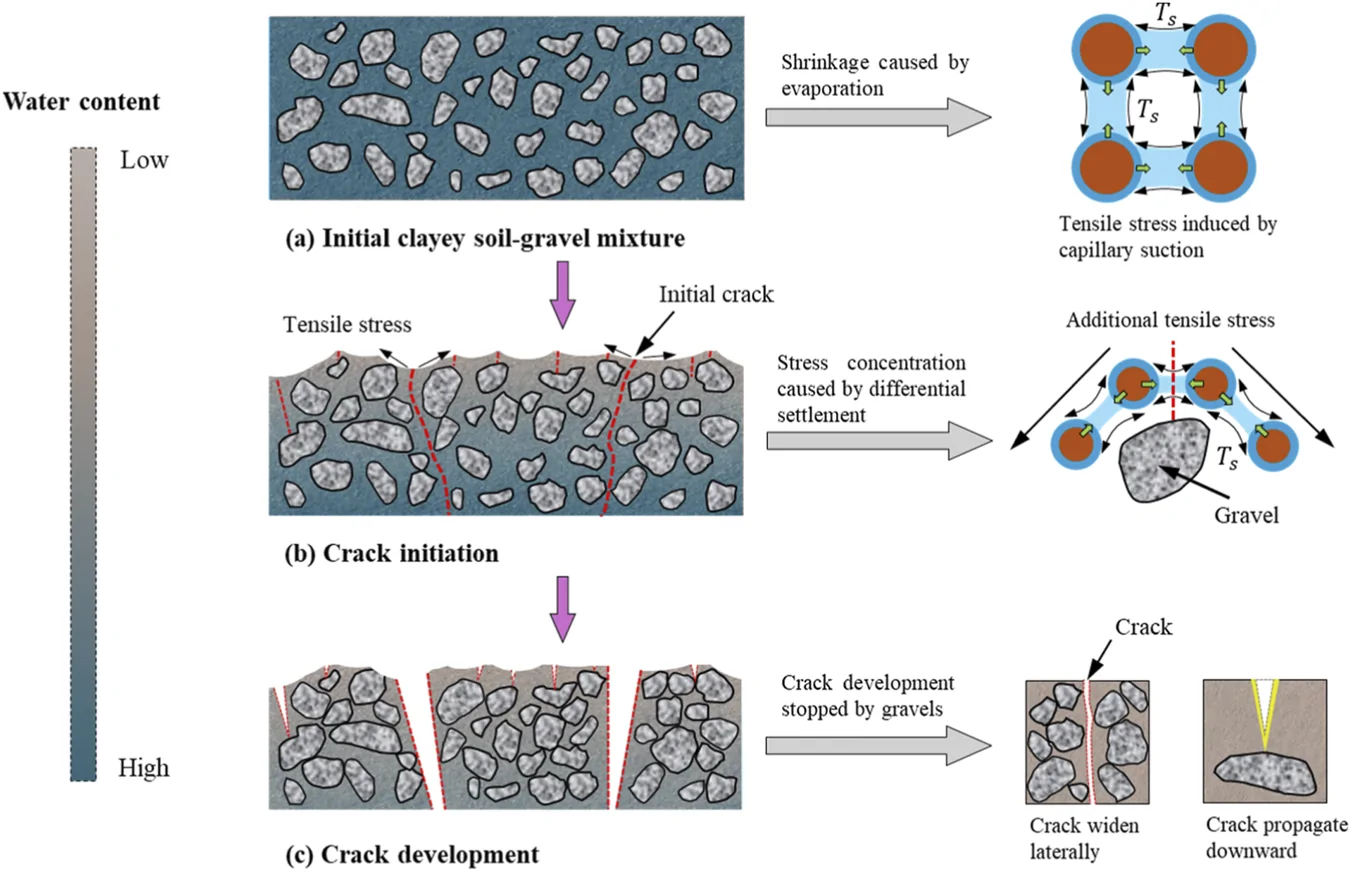
Effects of gravel on the crack initiation and development: **(a)** Initial clayey soil-gravel mixture; **(b)** Crack initiation; **(c)** Crack development.

It should be noted that the clayey soil–gravel mixture contains a lower volumetric clay fraction (approximately 60%) than the pure clay specimen, which reduces the total amount of shrinkable material. This could contribute to the observed lower final surface crack ratio. However, the earlier crack initiation and the higher number of crack segments in the mixture cannot be explained solely by the reduced clay content; a lower clay volume alone would tend to delay and reduce cracking if other factors were equal. Instead, these observations indicate that gravel inclusions create additional stress concentrations and physically restrain local shrinkage, leading to a denser but narrower crack network. The separate contributions of reduced clay volume and gravel-induced mechanical effects remain an important topic for future studies with varying gravel contents.

### 4.3 Influence on water retention and flow behavior

The surface crack length per unit area ( $L_{pa}$ ) and the number of crack segments per unit area ( $N_{pa}$ ) together describe not only the geometric density of the crack network but also its connectivity and tortuosity. A higher $L_{pa}$ indicates a greater total length of preferential flow paths per unit area, while a higher $N_{pa}$ indicates that these paths are more segmented and interconnected. In the clayey soil–gravel mixture, the values of $L_{pa}$ (0.85 cm/cm^2^) and $N_{pa}$ (0.36 cm^-2^) are respectively 1.39 and 1.71 times those of pure clay (0.61 cm/cm^2^ and 0.21 cm^-2^). This means that although the total surface area occupied by cracks ( $R_{C}$ ) is smaller in the mixture, the crack network is denser and more tortuous.

These morphological differences have direct implications for water retention and flow. Under wet, near-saturated conditions, the denser and more interconnected crack network can act as a system of preferential flow pathways. Even if individual cracks are narrower, their greater total length and connectivity can enhance near-saturated hydraulic conductivity and, in many cases, saturated hydraulic conductivity (Rayhani et al., 2007; Cheng et al., 2021); however, the saturated conductivity also depends on the crack aperture distribution, because crack conductance scales strongly with aperture. When water-filled, these cracks bypass the low-permeability clay matrix, allowing rapid water movement. Furthermore, the transition from a few wide cracks to many narrower cracks alters the pore size distribution: the cracks represent large, non-capillary pores that drain at low suction, thereby reducing the water content retained under low-suction, near-saturated conditions (Li et al., 2011). This results in a dual-porosity system in which the crack network controls preferential flow and early-time infiltration under wet conditions, while the clay matrix controls water retention at higher suctions (Luo et al., 2025). Consequently, $L_{pa}$ and $N_{pa}$ serve as morphological indicators of the degree to which gravel-induced cracking transforms the soil from a matrix-dominated flow system toward a crack-enhanced dual-porosity system.

## 5 Conclusion

Laboratory experiments were conducted on initially saturated specimen, including a pure clayey soil and a clayey soil-gravel mixture, to investigate the influence of gravel on cracking behavior of clayey soil. Using image processing techniques, several geometric parameters of the crack network at different water contents were determined. Additionally, the kinematic characteristics of surface crack patterns during drying were quantitatively described. The influence on evaporation rate, air entry value, desiccation crack initiation and development, cracking water content, surface crack ratio, and crack width was discussed. The following conclusions were drawn:

1. The presence of gravel within the clayey soil reduces the evaporation rate. Furthermore, it causes the saturated clayey soil-gravel mixture to become unsaturated at a higher overall water content compared to pure clayey soil. These effects are achieved by extending the travel distance and reducing the effective cross-sectional area for moisture moving upward.
2. The tensile stress in the clayey soil-gravel mixture originates from two primary sources: capillary suction due to meniscus formation and an additional component induced by differential settlement. Gravels can create more small protrusions on the soil surface, change the position of desiccation crack initiation and leads to earlier cracking.
3. Gravels can reduce the cracks’ width significantly by stop cracks propagating downward and limiting the block shrinkage. This is the main reason why the surface crack ratio decreases after adding gravels in clayey soil, even though the cracks grow slightly longer.

## Data Availability

The raw data supporting the conclusions of this article will be made available by the authors, without undue reservation.
